# Construction of an Immune-Associated Gene-Based Signature in Muscle-Invasive Bladder Cancer

**DOI:** 10.1155/2020/8866730

**Published:** 2020-12-29

**Authors:** Chengquan Shen, Ting Xu, Yefeng Sun, Liping Wang, Zhijuan Liang, Haitao Niu, Wei Jiao, Yonghua Wang

**Affiliations:** ^1^Department of Urology, The Affiliated Hospital of Qingdao University, Qingdao, Shandong, China; ^2^Department of Geratology, The 971st Hospital of PLA, Qingdao, Shandong, China; ^3^Key Laboratory of Urology and Andrology, The Affiliated Hospital of Qingdao University, Qingdao, Shandong, China

## Abstract

**Background:**

In recent years, immune-associated genes (IAGs) have been documented as having critical roles in the occurrence and progression of muscle-invasive bladder cancer (MIBC). Novel immune-related biomarkers and a robust prognostic signature for MIBC patients are still limited. The study is aimed at developing an IAG-based signature to predict the prognosis of MIBC patients.

**Methods:**

In the present study, we identified differentially expressed IAGs in MIBC by using transcriptomics data from The Cancer Genome Atlas (TCGA) database and proteomics data from our samples. We further constructed an IAG-based signature and evaluated its prognostic and predictive value by survival analysis and nomogram. Tumor Immune Estimation Resource (TIMER) was applied to explore the correlation between the IAG-based signature and immune cell infiltration in the microenvironment of MIBC.

**Results:**

A total of 22 differentially expressed IAGs were identified, and 2 IAGs (*NR2F6* and *AHNAK*) were used to establish a prognostic signature. Subsequently, survival analysis showed that high-risk scores were significantly correlated with poor overall survival (OS), progression-free survival (PFS), and disease-free survival (DFS) of MIBC patients. A prognostic nomogram was constructed by integrating clinical factors with the IAG-based signature risk score. In addition, the IAG-based signature risk score was positively associated with the infiltration of macrophages and dendritic cells in MIBC.

**Conclusions:**

We constructed and verified a novel IAG-based signature, which could predict the prognosis of MIBC and might reflect the status of the immune microenvironment of MIBC. Further studies in more independent clinical cohorts and further experimental exploration of the prognostic IAG-based signature are still needed.

## 1. Introduction

Bladder cancer (BC), a complex tumor associated with high morbidity and mortality rates in the urinary system, is the ninth most common malignant disease worldwide [1]. Approximately 25% of patients are diagnosed with muscle-invasive bladder cancer (MIBC), which is a potentially lethal malignancy with an inferior prognosis [2]. The primary treatment for MIBC is surgery, whereas immunotherapies are emerging as a preferred treatment for MIBC patients in whom surgery and chemotherapy cannot control the disease [3]. However, due to the intrinsic genetic heterogeneity of MIBC patients, patients with similar pathological features still have a different response rate to immunotherapies. At present, the precise molecular mechanisms of MIBC have not yet been realized, while a study demonstrated that genetic factors, especially immune-associated genes (IAGs) and immune cells, play important roles in the occurrence and progression of MIBC [4]. Furthermore, new advances in genome sequencing technology and bioinformatics have contributed to screening potential biomarkers that can predict the prognosis of cancer patients [5]. In recent years, The Cancer Genome Atlas (TCGA) database has been widely used to identify the differentially expressed genes in the mRNA expression profiles. In addition, a label-free liquid chromatography-tandem mass spectrometry- (LC-MS/MS-) based proteome profiling approach has been used to survey the expression levels of protein in samples [6]. Hence, novel immune-related biomarkers for predicting MIBC patient survival outcomes and immune status could be well studied by integrating with transcriptomics and proteomics data.

In the present study, transcriptomics data from the TCGA database and proteomics data from our samples were applied to identify differentially expressed IAGs in MIBC patients. A prognostic IAG-based signature and a nomogram were further constructed to predict the prognosis of MIBC patients. New potential prognostic markers in our study also provide preliminary bioinformatic evidence for understanding the complex mechanism of MIBC progression.

## 2. Materials

### 2.1. Data Collection

The raw RNA sequencing expression profile and corresponding clinical information of BC patients were obtained from the TCGA official website (https://portal.gdc.cancer.gov/repository). We further selected 164 MIBC patients with complete histopathologic information from BC patients, and the detailed clinical information of MIBC patients is shown in Table 1. An IAG comprehensive list is downloaded from the ImmPort database (https://immport.nia/http://id.nih.gov/) [7], which contains a total of 2498 IAGs.

### 2.2. LC-MS/MS

In the present study, all of the tissue samples were collected from 10 patients treated with surgical resection, including 10 MIBC tissues and corresponding normal tissues. The detailed clinical information of these 10 MIBC patients is shown in Table 1. These 10 patients underwent laparoscopic radical cystectomy and did not receive preoperative radiotherapy and chemotherapy. According to the ethical guidelines as required by the Declaration of Helsinki, informed consent was provided by each patient, and the research protocol was approved by the Ethical Committee of the Affiliated Hospital of Qingdao University [8].

Comparative proteomic profiling is commonly used in LC-MS/MS [9]. In this study, the same method was performed to characterize the variety of proteins in MIBC samples and normal samples. The process contained protein extraction, trypsin digestion, TMT/iTRAQ labeling, HPLC fractionation, LC-MS/MS analysis, database search, and bioinformatic analysis. The enrichment of the differentially expressed protein against all identified proteins was detected by two-tailed Fisher's exact test, and protein domains with a corrected *p* value < 0.05 were recognized as statistically significant. The threshold of the tumor/normal ratio was set as 1.2 to further distinguish up- or downregulation of these proteins in MIBC.

### 2.3. Identification of Differentially Expressed IAGs

The differentially expressed IAGs between TCGA MIBC and normal samples were identified using edgeR (version R 3.5.1, https://bioconductor.org/packages/release/bioc/) [10]. A false discovery rate (FDR) < 0.05 and ∣log2 fold change (FC) | >1 were set as the cut-off values. Subsequently, proteomics data were used to screen IAGs, which were differentially expressed in both mRNA and protein levels. A boxplot was applied to display these differentially expressed IAGs. Gene Ontology (GO) enrichment analysis and KEGG pathway analysis for differentially expressed IAGs were also performed using the “clusterProfiler” R package.

### 2.4. Construction of a Prognostic IAG-Based Signature

The univariate Cox regression analysis was performed to identify IAGs that were closely related to overall survival (OS) of the TCGA MIBC cohort. The hazard ratios (HRs) were used to identify risk-related IAGs (HR > 1) and protective IAGs (HR < 1). The prognostic IAGs as the candidate genes were subjected to a least absolute shrinkage and selection operator (LASSO) Cox regression to construct an optimal IAG-based signature for predicting the prognosis of MIBC patients. The prognostic risk score was computed for each MIBC patient using the following formula: risk score = ∑(*βi* × Exp*i*) (*i* = the number of prognostic IAGs) [11]. After calculating the risk scores of MIBC patients, 164 MIBC patients were divided into high- and low-risk groups according to the median value of the risk score. A Kaplan-Meier (K-M) curve was constructed to assess the survival outcome difference between high- and low-risk groups. A receiver operating characteristic (ROC) curve was conducted to evaluate the predictive accuracy of clinicopathologic characteristics and the IAG signature with the R package “survivalROC.” Furthermore, univariate and multivariate Cox regression analyses were performed to assess whether the risk score was independent of other clinical variables such as age, gender, grade, stage, pathologic T, pathologic N, pathologic M, and histological subtype in determining the prognosis of the MIBC patients. Moreover, 164 MIBC patients were clustered into two molecular subtypes (basal and luminal) based on the expression levels of genes [12]. The expressed levels of two IAGs in two molecular subtypes were analyzed by using the Wilcoxon signed-rank test.

### 2.5. Construction of a Prognostic Nomogram

A nomogram was constructed by integrating clinical variables (age, gender, grade, pathologic stage, pathologic T, pathologic N, pathologic M, and subtype) and the risk score derived from the prognostic signature to assess the probable 1-, 2-, and 3-year OS of MIBC patients via the R package rms (https://cran.r-project.org/web/packages/rms/) [13].

### 2.6. Correlation Analysis between Risk Score and Immune Cell Infiltration in MIBC

Previous studies demonstrated that tumor-infiltrating immune cells, such as macrophages, B cells, and CD8+ T cells, can influence the balance between antitumor immunity and immune evasion in MIBC [14–16]. Thus, Tumor Immune Estimation Resource (TIMER), a useful resource for comprehensive analysis of tumor-infiltrating immune cells, was employed to explore the correlations between the signature risk score and immune cell infiltration [17]. The composition of six tumor-infiltrating immune cell subsets (B cells, CD4+ T cells, CD8+ T cells, macrophages, neutrophils, and dendritic cells) was estimated by using the TIMER algorithm. The levels of immune cell infiltration in MIBC patients were obtained from the TIMER website, and the relationship between the signature risk score and six tumor-infiltrating immune cells was performed in R.

## 3. Results

### 3.1. Identification of Differentially Expressed IAGs in MIBC

**Figure 1:**
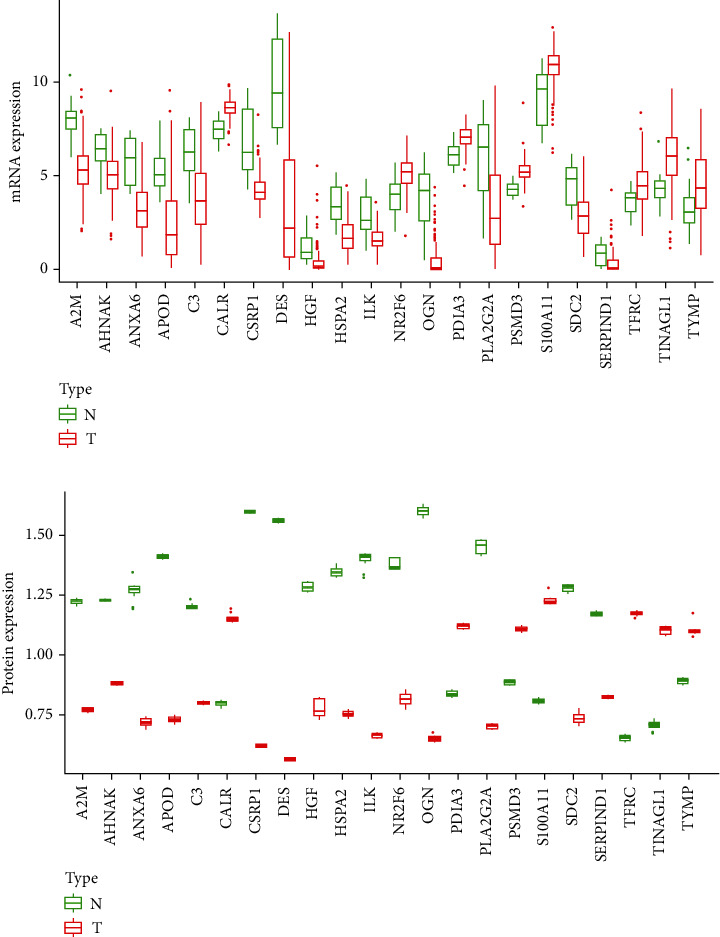
Boxplots of the mRNA (a) and protein (b) expression levels of 22 IAGs in MIBC and normal tissues.

**Figure 2:**
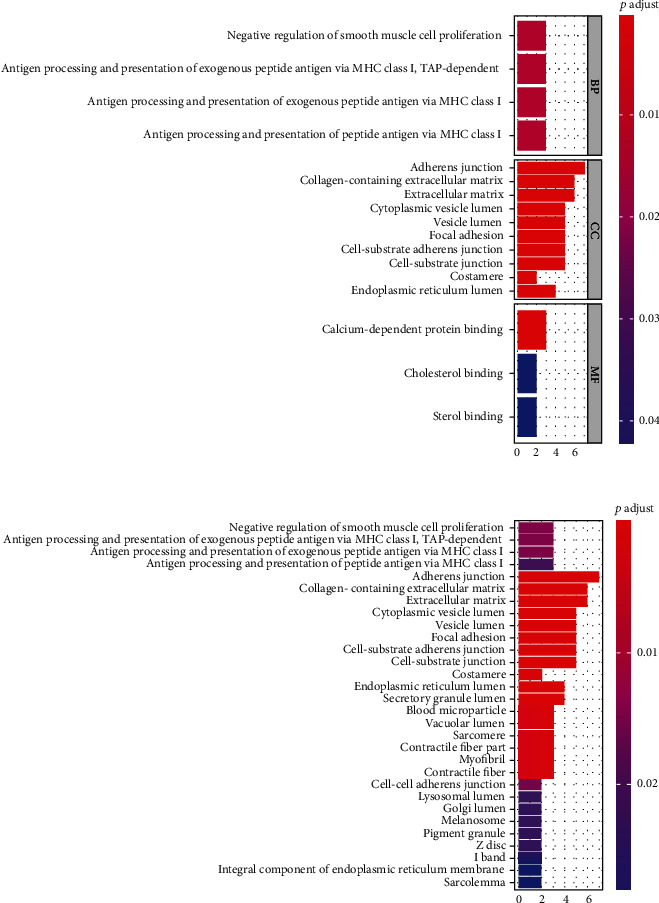
GO and KEGG analyses of differentially expressed IAGs. Heatmap exhibited the enriched GO terms across the differentially expressed IAGs (a). Heatmap exhibited the enriched KEGG pathways across the differentially expressed IAGs (b).

The transcriptomics data and proteomics data of MIBC patients were subjected to differential expression analysis. A total of 22 differentially expressed IAGs were identified in MIBC samples. Boxplots were used to display the expression levels of 22 IAGs. Among 22 IAGs, 14 IAGs were downregulated and 8 IAGs were upregulated in TCGA MIBC samples (Figure 1(a)). In our samples, 15 IAGs were downregulated and 7 IAGs were upregulated (Figure 1(b)). The subcellular localization of 22 IAGs in tumor cells is mainly enriched in the cytoplasm, endoplasmic reticulum, extracellular, mitochondria, nucleus, and plasma membrane (Supplementary 1). To investigate the potential molecular mechanisms of 22 IAGs in MIBC, GO and KEGG analyses were performed. The results indicated that the most significant GO enriched terms involved in immunity were negative regulation of smooth muscle cell proliferation and antigen processing and presentation of exogenous peptide antigen via MHC class I (BP: biological process); adherens junction, collagen-containing extracellular matrix, and extracellular matrix (CC: cellular components); and calcium-dependent protein binding (MF: molecular functions) (Figure 2(a)). In the KEGG enrichment analysis, these IAGs were primarily correlated with pathways related to adherens junction, extracellular matrix, vesicle lumen, focal adhesion, cell-substrate junction, and costamere (Figure 2(b)).

### 3.2. Construction of an IAG-Based Signature

**Figure 3:**
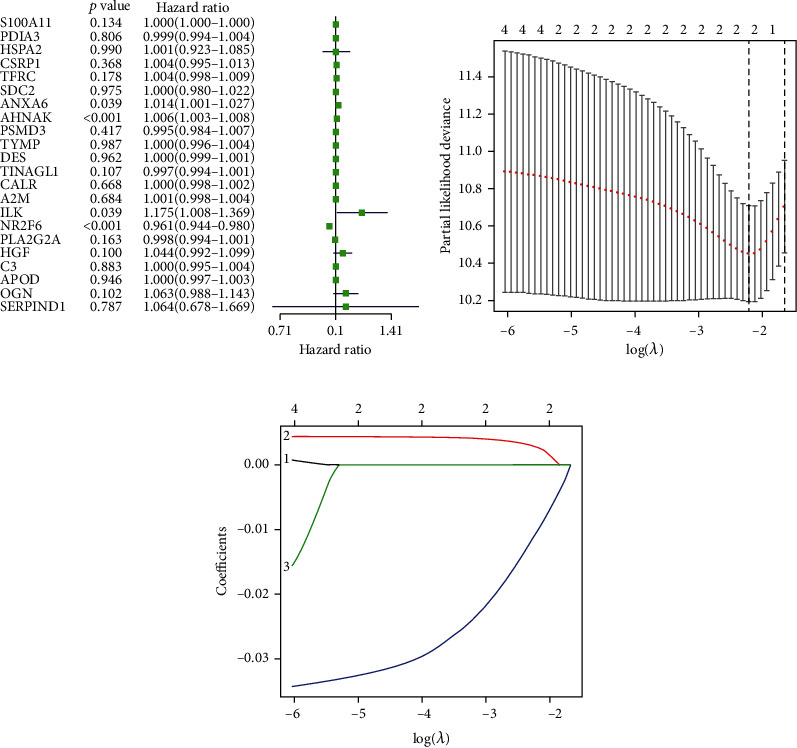
Univariate Cox regression analysis showed that a total of 4 IAGs are closely associated with the survival of MIBC patients (a). LASSO coefficient profiles of 4 genes in MIBC. Selection of the optimal parameter (lambda) in the LASSO model for MIBC (b). A coefficient profile plot was generated against the log(lambda) sequence (c).

**Figure 4:**
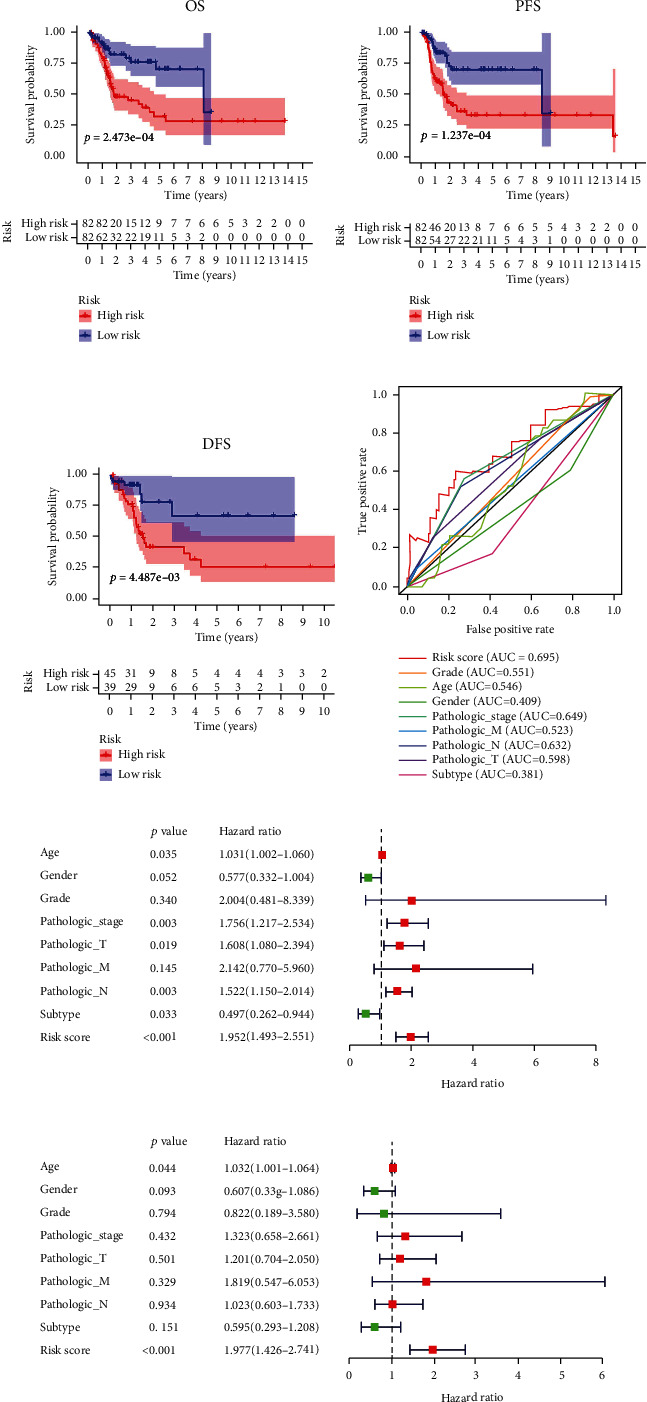
Survival analyses of the IAG-based signature in MIBC. Kaplan-Meier curves revealed that the high-risk score was closely associated with poor overall survival (OS) (a), progression-free survival (PFS) of MIBC patients (b), and disease-free survival (DFS) of MIBC patients (c). The ROC curves showed that AUCs for the signature risk score, grade, age, gender, pathologic stage, pathologic M, pathologic N, pathologic T, and subtype were 0.695, 0.551, 0.546, 0.409, 0.649, 0.523, 0.632, 0.598, and 0.381, respectively (d). Univariate Cox regression analysis showed that age, pathologic stage, pathologic T, pathologic M, subtype, and risk score were associated with the OS of MIBC patients (e). Multivariate Cox regression analysis indicated that the IAG-based signature risk score was an independent prognostic factor for MIBC (f).

**Figure 5:**
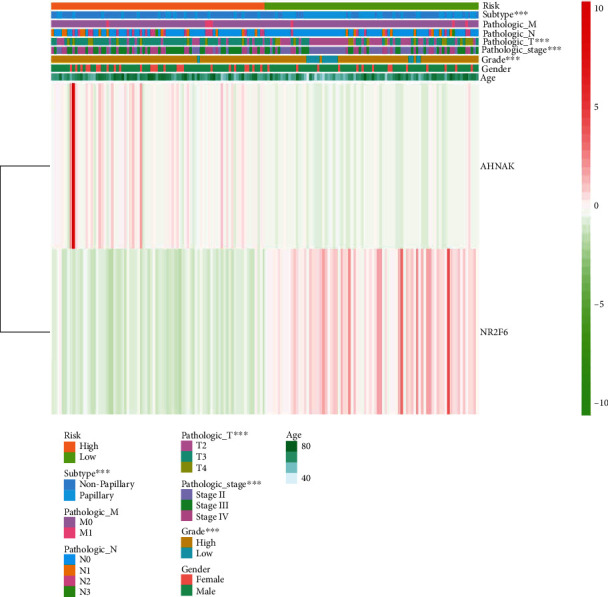
Correlation analysis of the IAG-based signature with other clinicopathological parameters. Heatmap indicated that the signature was significantly correlated with the grade, pathologic stage, pathologic T, and subtype of MIBC. ^∗^*p* < 0.05; ^∗∗^*p* < 0.01; ^∗∗∗^*p* < 0.001.

**Figure 6:**
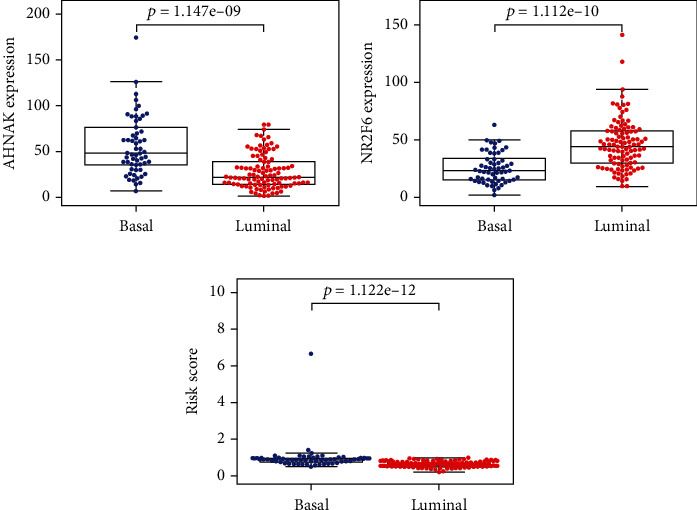
The expression level of *AHNAK* and *NR2F6* in different molecular subtypes of BC. The results indicated that *AHNAK* was highly expressed in the basal subtype and *NR2F6* was highly expressed in the luminal subtype (a, b). Furthermore, MIBC patients with a high IAG-based signature risk score may indicate the feature of a basal subtype (c).

By performing univariate Cox regression analysis on the 22 IAGs in the TCGA cohort, *ANXA6* (annexin A6), *AHNAK* (AHNAK nucleoprotein), *ILK* (integrin-linked kinase), and *NR2F6* (nuclear receptor subfamily 2 group F member 6) were significantly related to the OS of MIBC patients (Figure 3(a)). Among 4 IAGs, *ANXA6*, *AHNAK*, and *ILK* were considered risk factors with HR values greater than 1, whereas *NR2F6* was considered a protective factor with HR values less than 1. LASSO Cox regression analysis was then applied to construct an IAG-based signature in MIBC by using 4 IAGs. While 2 genes were included, the signature achieved the best performance (Figure 3(b)), and the regression coefficient for 4 IAGs was computed (Figure 3(c)). The risk score for each MIBC patient is as follows: risk score = (0.0009 × expression value of *AHNAK*) + (−0.0060 × expression value of *NR*2*F*6), and classified 164 patients into high- and low-risk groups according to the median value of the risk score. K-M survival analysis demonstrated that high-risk scores were significantly associated with poor OS (*p* = 2.473*e* − 04, Figure 4(a)), progression-free survival (PFS) (*p* = 1.237*e* − 04, Figure 4(b)), and disease-free survival (DFS) (*p* = 4.487*e* − 03, Figure 4(c)). To further evaluate the predictive ability of the IAG signature, we performed the ROC curve, and the area under the curve (AUC) of the signature risk score, grade, age, gender, pathologic stage, pathologic M, pathologic N, pathologic T, and subtype were 0.695, 0.551, 0.546, 0.409, 0.649, 0.523, 0.632, 0.598, and 0.381, respectively (Figure 4(d)). The result indicated superior predictive accuracy of the signature in survival than other clinical factors. Subsequently, univariate and multivariate Cox regression analyses were performed to assess the prognostic significances of the IAG-based signature and various clinical factors. Univariate Cox regression analysis showed that age (*p* = 0.035), pathologic stage (*p* = 0.003), pathologic T (*p* = 0.019), pathologic M (*p* = 0.003), subtype (*p* = 0.033), and risk score (*p* < 0.001) were associated with the OS of MIBC patients (Figure 4(e)). Multivariate Cox regression analysis indicated that the IAG signature risk score was an independent prognostic factor for MIBC (*p* < 0.001, Figure 4(f)). In addition, the correlation of the IAG-based signature and the clinicopathological characteristics of MIBC patients was also analyzed. A heatmap indicated that the signature was closely associated with the grade (*p* < 0.001), pathologic stage (*p* < 0.001), pathologic T (*p* < 0.001), and subtype (*p* < 0.001) of MIBC patients (Figure 5). In recent years, several independent studies have shown that BC has distinct molecular subtypes, which were associated with different outcomes of BC patients. Therefore, the expression levels of two IAGs in two molecular types were further analyzed. The results indicated that *AHNAK* was highly expressed in the basal subtype and *NR2F6* was highly expressed in the luminal subtype (Figures 6(a) and 6(b)). Furthermore, MIBC patients with a high IAG-based signature risk score may indicate the features of a basal subtype (Figure 6(c)).

### 3.3. Construction of a Prognostic Nomogram for MIBC

**Figure 7:**
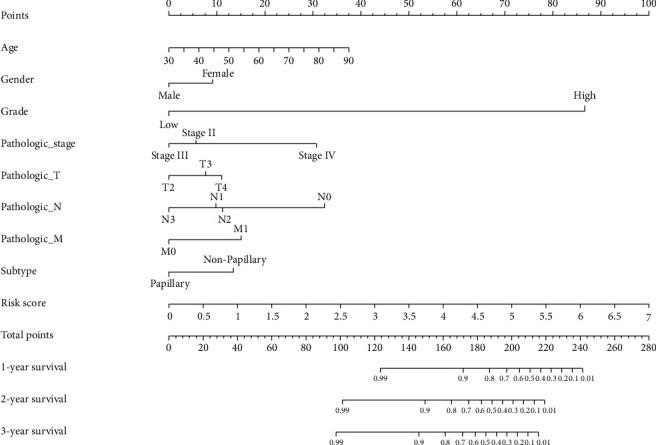
The prognostic nomogram with clinicopathological characteristics and the IAG signature risk score was developed from the TCGA MIBC cohort. The nomogram could predict the 1-, 2-, and 3-year survival probability of MIBC patients.

To establish a clinically applicable method for monitoring the prognosis of MIBC patients, we established a prognostic nomogram by combining clinicopathologic characteristics (age, gender, grade, pathologic stage, pathologic T, pathologic N, pathologic M, and subtype) with the signature risk score. The result indicated that the new prognostic nomogram could superiorly predict 1-, 2-, and 3-year OS of MIBC patients (Figure 7).

### 3.4. Correlation Analysis between the Risk Score and Immune Cell Infiltration in MIBC

**Figure 8:**
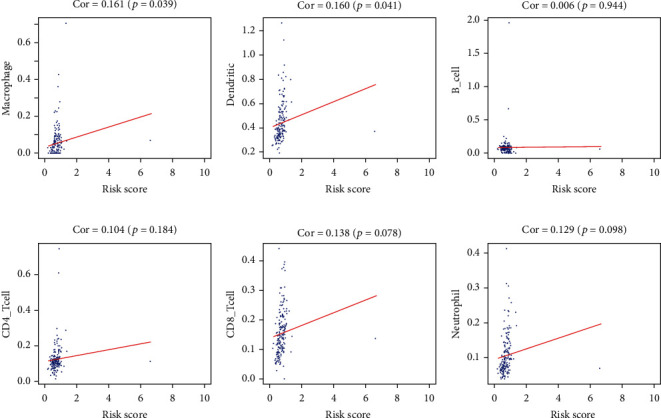
Association between the IAG signature risk score and immune cell infiltration in the microenvironment of MIBC. The results revealed that the risk score was positively related to the infiltration of macrophages (a) and dendritic cells (b). However, the risk score was not correlated with the infiltration of B cells, CD4+ T cells, CD8+ T cells, and neutrophils (c–f).

We estimated the relationship between the abundance of six types of tumor-infiltrating immune cells (B cells, CD4+ T cells, CD8+ T cells, dendritic cells, macrophages, and neutrophils) and the signature risk score in MIBC. The results revealed that the risk score was positively related to the infiltration of macrophages (*p* = 0.039, Figure 8(a)) and dendritic cells (*p* = 0.041, Figure 8(b)) in MIBC. However, the risk score was not correlated with the infiltration of B cells, CD4+ T cells, CD8+ T cells, and neutrophils (Figures 8(c)–8(f)).

## 4. Discussion

MIBC is a common malignancy of the urinary system, in which incidence rates are constantly increasing worldwide. Due to the profound research on the mechanism of tumor immune escape and immunotherapy, IAGs are getting increasing attention in recent years. Current studies had shown the important role of IAGs in various cancers, including MIBC [18–21]. Therefore, IAGs are novel biomarkers, which can predict the survival outcomes and the immune status of MIBC patients. In the present study, 22 IAGs were identified based on the transcriptomics and proteomics data of MIBC patients; 4 IAGs (*ANXA6*, *AHNAK*, *ILK*, and *NR2F6*) were significantly related to the survival of MIBC patients. Subsequently, *AHNAK* and *NR2F6* were finally selected to construct a signature to predict the prognosis of MIBC patients. Survival analysis showed that the signature was associated with the OS, PFS, and DFS of MIBC patients and can serve as an independent factor in predicting the survival of MIBC patients. In addition, a significant difference was found between the high- and low-risk groups for the molecular subtype. Therefore, MIBC patients with a basal molecular type may indicate high-risk scores and poor survival outcomes. Previous studies also demonstrated that the expression of IAGs, such as LAG3, PD-L1, CD3, and IL22, was closely related to the prognosis of different subtypes of MIBC [21–23]. Furthermore, a prognostic nomogram integrated with the IAG signature risk score and clinicopathologic features was established, which could superiorly monitor the prognosis of MIBC patients. Previous studies showed that immune cells were correlated with the prognosis, metastasis, and immune escape of cancers [24–26]. Therefore, we estimated the relationship between the abundance of six types of tumor-infiltrating immune cells (B cells, CD4+ T cells, CD8+ T cells, neutrophils, macrophages, and dendritic cells) and the signature risk score in MIBC. The result revealed that the risk score was positively related to the infiltration of macrophages and dendritic cells. Macrophages, especially M2 tumor-associated macrophages, can promote the invasion, metastasis, carcinogenesis of cancers, and dendritic cells that can mediate the antitumor immune response of BC [27–29]. Thus, *AHNAK* and *NR2F6* may promote the progression of MIBC by mediating the number of macrophages and dendritic cells in the microenvironment of MIBC.

It is worth noting that these two IAGs had participated in different immune pathways to influence the prognosis of tumors. *NR2F6*, an orphan nuclear receptor, regulates various biological and embryological processes, such as cellular differentiation and organogenesis [30, 31]. In immune cells, it contributes to regulating the expression of cytokines and it serves as a negative regulator in the development of T cells [32–34]. In addition, *NR2F6* was associated with the expression of programmed cell death-1 (PD-1), programmed cell death ligand-1 (PD-L1), and cytotoxic T lymphocyte-associated protein 4 (CTLA-4), and it was considered a potential target for immunotherapy [35–37]. *NR2F6* was also defined as an intracellular immune checkpoint in tumor-infiltrating T cells and is significantly related to the survival outcomes of numerous cancers, such as head and neck squamous cell carcinoma, early-stage cervical cancer, and colon cancer [38–40]. Furthermore, overexpression of *NR2F6* can promote the chemoresistance of epithelial ovarian cancer by activating the Notch3 signaling pathway [41]. *AHNAK*, also known as desmoyokin [42], is a large protein that was originally identified as a desmosomal plaque protein found at the periphery of the cytoplasmic plaque of desmosomes in the stratified squamous epithelia [43]. *AHNAK* has been previously reported to be expressed in several intracellular locations, including the plasma membrane, cytoplasm, and nucleus [44]. Previous studies have indicated that *AHNAK* has participated in several important physiological activities, such as cardiac L-type Ca^2+^ channel function [45], neuronal cell differentiation, and calcium signaling in T cells [46, 47]. In recent years, it has been demonstrated that *AHNAK* serves as a novel prognostic biomarker in different types of cancer, such as pancreatic ductal adenocarcinoma, triple-negative breast cancer, and BC [48–50]. Moreover, *AHNAK* also correlated with the progression, migration, and invasion of cancers [51–53]. Sohn et al. showed that AHNAK can promote the metastasis of tumors through transforming growth factor-*β*-mediated epithelial-mesenchymal transition [54]. However, a study suggested that *AHNAK* can act as a tumor suppressor that mediates the negative regulation of cell growth via the modulation of the TGF*β*/Smad signaling pathway [55]. Therefore, the precise molecular mechanisms of *AHNAK* in MIBC still needed to be elucidated. In our study, the AUC of the signature is 0.695, and the signature was positively related to the infiltration of macrophages and dendritic cells in MIBC. Therefore, we suggested that the IAG signature could not only predict the prognosis of MIBC patients but also reflect the immune status of MIBC patients. However, there are several limitations to our research. Firstly, this is a retrospective study. Therefore, we could not obtain the complete information of MIBC patients. Secondly, our findings need to be further validated in other independent cohorts to validate the robustness of the IAG-based signature. Thirdly, all the analyses are descriptive, and further experimental studies are needed to investigate the potential role of these IAGs in predicting the prognosis, especially response to immunotherapy for MIBC.

In conclusion, we established and verified a novel IAG-based signature that could reflect the prognosis of MIBC based on transcriptomics and proteomics data and might reflect the status of the immune microenvironment of MIBC patients. Further studies in more independent clinical cohorts and further experimental exploration of the prognostic IAGs signature are needed.

## Figures and Tables

**Table 1:** TCGA and our MIBC patient characteristics.

Clinical characteristics		TCGA (*n* = 164)	%	Our samples (*n* = 10)	%
Survival status	Alive	107	65.24	9	90
	Dead	57	34.76	1	10

Age at diagnosis		68 (34-87)		64 (48-77)	

Gender	Female	39	23.78	2	20
	Male	125	76.22	8	80

Histologic grade	High grade	148	90.24	5	50
	Low grade	16	9.76	5	50

Stage	I	0	0	0	0
	II	49	29.88	2	20
	III	63	38.41	4	40
	IV	52	31.71	4	40

Pathologic_T	T1	0	0	0	0
	T2	55	33.54	2	20
	T3	86	52.44	7	70
	T4	23	14.02	1	10

Pathologic_M	M0	157	95.73	9	90
	M1	7	4.27	1	1

Pathologic_N	N0	115	70.12	8	80
	N1	20	12.20	1	10
	N2	27	16.46	1	10
	N3	2	1.22	0	0

Histological subtype	Nonpapillary	103	62.8	2	20
	Papillary	61	37.2	8	80

## Data Availability

The data used to support the findings of this study is included within the article, and the data are available from the corresponding authors upon request.

## Supplementary Materials

Supplementary Materials Supplementary 1: the differences in the protein levels of IAGs between MIBC samples and normal samples.

## References

[1] Antoni S., Ferlay J., Soerjomataram I., Znaor A., Jemal A., Bray F. (2017). Bladder cancer incidence and mortality: a global overview and recent trends. *European Urology*.

[2] Smith A. B., Deal A. M., Woods M. E. (2014). Muscle-invasive bladder cancer: evaluating treatment and survival in the National Cancer Data Base. *BJU International*.

[3] Rijnders M., de Wit R., Boormans J. L., Lolkema M. P. J., van der Veldt A. A. M. (2017). Systematic review of immune checkpoint inhibition in urological cancers. *European Urology*.

[4] Schneider A. K., Chevalier M. F., Derré L. (2019). The multifaceted immune regulation of bladder cancer. *Nature Reviews. Urology*.

[5] Huang D. W., Sherman B. T., Lempicki R. A. (2009). Bioinformatics enrichment tools: paths toward the comprehensive functional analysis of large gene lists. *Nucleic Acids Research*.

[6] Bahrami S., Gheysarzadeh A., Sotoudeh M. (2020). The association between gelsolin-like actin-capping protein (Cap G) overexpression and bladder cancer prognosis. *Urology Journal*.

[7] Bhattacharya S., Andorf S., Gomes L. (2014). ImmPort: disseminating data to the public for the future of immunology. *Immunologic Research*.

[8] Shen C., Liu J., Wang L., Liang Z., Niu H., Wang Y. (2020). Identification of metabolism-associated genes and construction of a prognostic signature in bladder cancer. *Cancer Cell International*.

[9] Stroggilos R., Mokou M., Latosinska A. (2020). Proteome-based classification of nonmuscle invasive bladder cancer. *International Journal of Cancer*.

[10] Robinson M. D., McCarthy D. J., Smyth G. K. (2010). edge R: a bioconductor package for differential expression analysis of digital gene expression data. *Bioinformatics*.

[11] Shen C., Liu J., Wang J. (2020). Development and validation of a prognostic immune-associated gene signature in clear cell renal cell carcinoma. *International Immunopharmacology*.

[12] Robertson A. G., Kim J., Al-Ahmadie H. (2017). Comprehensive molecular characterization of muscle-invasive bladder cancer. *Cell*.

[13] Qian Z., Li Y., Fan X. (2018). Prognostic value of a microRNA signature as a novel biomarker in patients with lower-grade gliomas. *Journal of Neuro-Oncology*.

[14] Zhou L., Xu L., Chen L. (2017). Tumor-infiltrating neutrophils predict benefit from adjuvant chemotherapy in patients with muscle invasive bladder cancer. *Oncoimmunology*.

[15] Jiang Q., Fu Q., Chang Y. (2019). CD19^+^ tumor-infiltrating B-cells prime CD4^+^ T-cell immunity and predict platinum-based chemotherapy efficacy in muscle-invasive bladder cancer. *Cancer Immunology, Immunotherapy*.

[16] Vesely M. D., Kershaw M. H., Schreiber R. D., Smyth M. J. (2011). Natural innate and adaptive immunity to cancer. *Annual Review of Immunology*.

[17] Li T., Fan J., Wang B. (2017). TIMER: a web server for comprehensive analysis of tumor-infiltrating immune cells. *Cancer Research*.

[18] Grant F. M., Yang J., Nasrallah R. (2020). BACH2 drives quiescence and maintenance of resting Treg cells to promote homeostasis and cancer immunosuppression. *The Journal of Experimental Medicine*.

[19] Du L., Lee J. H., Jiang H. (2020). *β*-Catenin induces transcriptional expression of PD-L1 to promote glioblastoma immune evasion. *The Journal of Experimental Medicine*.

[20] Zhou Q., Zhang H., Wang Z. (2020). Poor clinical outcomes and immunoevasive contexture in interleukin-9 abundant muscle-invasive bladder cancer. *International Journal of Cancer*.

[21] Zeng H., Zhou Q., Wang Z. (2020). Stromal LAG-3^+^ cells infiltration defines poor prognosis subtype muscle-invasive bladder cancer with immunoevasive contexture. *Journal for Immunotherapy of Cancer*.

[22] Li H., Zhang Q., Shuman L. (2020). Evaluation of PD-L1 and other immune markers in bladder urothelial carcinoma stratified by histologic variants and molecular subtypes. *Scientific Reports*.

[23] Zeng H., Liu Z., Wang Z. (2020). Intratumoral IL22-producing cells define immunoevasive subtype muscle-invasive bladder cancer with poor prognosis and superior nivolumab responses. *International Journal of Cancer*.

[24] Wang X., Duanmu J., Fu X., Li T., Jiang Q. (2020). Analyzing and validating the prognostic value and mechanism of colon cancer immune microenvironment. *Journal of Translational Medicine*.

[25] Flerin N. C., Pinioti S., Menga A., Castegna A., Mazzone M. (2020). Impact of immunometabolism on cancer metastasis: a focus on T cells and macrophages. *Cold Spring Harbor Perspectives in Medicine*.

[26] Grabovska Y., Mackay A., O'Hare P. (2020). Pediatric pan-central nervous system tumor analysis of immune-cell infiltration identifies correlates of antitumor immunity. *Nature Communications*.

[27] Wu H., Zhang X., Han D., Cao J., Tian J. (2020). Tumour-associated macrophages mediate the invasion and metastasis of bladder cancer cells through CXCL8. *Peer J*.

[28] Wu A. T. H., Srivastava P., Yadav V. K. (2020). Ovatodiolide, isolated from *Anisomeles indica*, suppresses bladder carcinogenesis through suppression of mTOR/*β*-catenin/CDK6 and exosomal miR-21 derived from M2 tumor-associated macrophages. *Toxicology and Applied Pharmacology*.

[29] Chevalier M. F., Bohner P., Pieraerts C. (2017). Immunoregulation of dendritic cell subsets by inhibitory receptors in urothelial cancer. *European Urology*.

[30] Tsai S. Y., Tsai M. J. (1997). Chick ovalbumin upstream promoter-transcription factors (COUP-TFs): coming of age. *Endocrine Reviews*.

[31] Takamoto N., You L. R., Moses K. (2005). COUP-TFII is essential for radial and anteroposterior patterning of the stomach. *Development*.

[32] Hermann-Kleiter N., Baier G. (2014). Orphan nuclear receptor NR2F6 acts as an essential gatekeeper of Th17 CD4^+^ T cell effector functions. *Cell Communication and Signaling: CCS*.

[33] Olson W. J., Jakic B., Labi V. (2019). Orphan nuclear receptor NR2F6 suppresses T follicular helper cell accumulation through regulation of IL-21. *Cell reports*.

[34] Ichim C. V., Dervović D. D., Zúñiga-Pflücker J. C., Wells R. A. (2014). The orphan nuclear receptor Ear-2 (Nr2f6) is a novel negative regulator of T cell development. *Experimental Hematology*.

[35] Klepsch V., Hermann-Kleiter N., Do-Dinh P. (2018). Nuclear receptor NR2F6 inhibition potentiates responses to PD-L1/PD-1 cancer immune checkpoint blockade. *Nature Communications*.

[36] Hermann-Kleiter N., Klepsch V., Wallner S. (2015). The nuclear orphan receptor NR2F6 is a central checkpoint for cancer immune surveillance. *Cell Reports*.

[37] Klepsch V., Pommermayr M., Humer D., Brigo N., Hermann-Kleiter N., Baier G. (2020). Targeting the orphan nuclear receptor NR2F6 in T cells primes tumors for immune checkpoint therapy. *Cell Communication and Signaling: CCS*.

[38] Klapper L., Ribbat-Idel J., Kuppler P. (2020). NR2F6 as a prognostic biomarker in HNSCC. *International Journal of Molecular Sciences*.

[39] Niu C., Sun X., Zhang W. (2016). NR2F6 expression correlates with pelvic lymph node metastasis and poor prognosis in early-stage cervical cancer. *International Journal of Molecular Sciences*.

[40] Li X. B., Jiao S., Sun H. (2011). The orphan nuclear receptor EAR2 is overexpressed in colorectal cancer and it regulates survivability of colon cancer cells. *Cancer Letters*.

[41] Li H., Zhang W., Niu C. (2019). Nuclear orphan receptor NR2F6 confers cisplatin resistance in epithelial ovarian cancer cells by activating the Notch 3 signaling pathway. *International Journal of Cancer*.

[42] Amagai M. (2004). A mystery of AHNAK/desmoyokin still goes on. *The Journal of Investigative Dermatology*.

[43] Hieda Y., Tsukita S., Tsukita S. (1989). A new high molecular mass protein showing unique localization in desmosomal plaque. *The Journal of Cell Biology*.

[44] de Morrée A., Droog M., Grand Moursel L. (2012). Self-regulated alternative splicing at the AHNAK locus. *The FASEB Journal*.

[45] Haase H. (2007). Ahnak, a new player in beta-adrenergic regulation of the cardiac L-type Ca^2+^ channel. *Cardiovascular Research*.

[46] Hohaus A., Person V., Behlke J., Schaper J., Morano I., Haase H. (2002). The carboxyl-terminal region of ahnak provides a link between cardiac L-type Ca2+ channels and the actin-based cytoskeleton. *The FASEB Journal*.

[47] Haase H., Podzuweit T., Lutsch G. (1999). Signaling from beta-adrenoceptor to L-type calcium channel: identification of a novel cardiac protein kinase A target possessing similarities to AHNAK. *The FASEB Journal*.

[48] Zhang Z., Liu X., Huang R., Liu X., Liang Z., Liu T. (2019). Upregulation of nucleoprotein AHNAK is associated with poor outcome of pancreatic ductal adenocarcinoma prognosis via mediating epithelial-mesenchymal transition. *Journal of Cancer*.

[49] Chen B., Wang J., Dai D. (2017). AHNAK suppresses tumour proliferation and invasion by targeting multiple pathways in triple-negative breast cancer. *Journal of Experimental & Clinical Cancer Research*.

[50] Lee H., Kim K., Woo J. (2018). Quantitative proteomic analysis identifies AHNAK (neuroblast differentiation-associated protein AHNAK) as a novel candidate biomarker for bladder urothelial carcinoma diagnosis by liquid-based cytology. *Molecular & Cellular Proteomics*.

[51] Liu Z. M., Yang X. L., Jiang F., Pan Y. C., Zhang L. (2020). Matrine involves in the progression of gastric cancer through inhibiting mi R-93-5p and upregulating the expression of target gene AHNAK. *Journal of Cellular Biochemistry*.

[52] Cho W. C., Jang J. E., Kim K. H., Yoo B. C., Ku J. L. (2020). SORBS1 serves a metastatic role via suppression of AHNAK in colorectal cancer cell lines. *International Journal of Oncology*.

[53] Shen E., Wang X., Liu X. (2020). MicroRNA-93-5p promotes epithelial-mesenchymal transition in gastric cancer by repressing tumor suppressor AHNAK expression. *Cancer Cell International*.

[54] Sohn M., Shin S., Yoo J. Y., Goh Y., Lee I. H., Bae Y. S. (2018). Ahnak promotes tumor metastasis through transforming growth factor-*β*-mediated epithelial-mesenchymal transition. *Scientific Reports*.

[55] Lee I. H., Sohn M., Lim H. J. (2014). Ahnak functions as a tumor suppressor via modulation of TGF*β*/Smad signaling pathway. *Oncogene*.

