# Supplementary material for: Construction of an Immune-Associated Gene-Based Signature in Muscle-Invasive Bladder Cancer
Source: Dis Markers. 2020 Dec 29;2020:8866730. doi: 10.1155/2020/8866730 (PMC7785346; doi:10.1155/2020/8866730)
Supplement: Supplementary Materials — Supplementary 1: the differences in the protein levels of IAGs between MIBC samples and normal samples. [file 8866730.f1.docx]

**Supplementary 1**  **The differences in the protein levels of IAGs between MIBC samples and normal samples.**

| Gene name | T/N Ratio | Regulated Type | T/N P value | Subcellular localization |
| --- | --- | --- | --- | --- |
| ANXA6 | 0.456 | Down | 3.401E-07 | cytoplasm |
| HSPA2 | 0.446 | Down | 1.6686E-06 | cytoplasm |
| ILK | 0.352 | Down | 1.1855E-07 | cytoplasm |
| PSMD3 | 1.36 | Up | 2.0066E-05 | cytoplasm |
| S100A11 | 1.781 | Up | 1.2988E-06 | cytoplasm |
| C3 | 0.571 | Down | 6.1782E-08 | endoplasmic reticulum |
| CALR | 1.638 | Up | 1.80503E-05 | endoplasmic reticulum |
| PDIA3 | 1.498 | Up | 1.56547E-05 | endoplasmic reticulum |
| A2M | 0.533 | Down | 1.0219E-06 | extracellular |
| APOD | 0.397 | Down | 4.869E-07 | extracellular |
| HGF | 0.506 | Down | 5.8604E-05 | extracellular |
| OGN | 0.283 | Down | 7.9662E-07 | extracellular |
| PLA2G2A | 0.362 | Down | 2.1436E-06 | extracellular |
| SDC2 | 0.468 | Down | 3.7945E-05 | extracellular |
| SERPIND1 | 0.612 | Down | 8.0969E-07 | extracellular |
| TINAGL1 | 1.822 | Up | 5.7418E-05 | extracellular |
| DES | 0.246 | Down | 6.9389E-09 | mitochondria |
| AHNAK | 0.631 | Down | 7.024E-08 | nucleus |
| CSRP1 | 0.265 | Down | 1.6684E-09 | nucleus |
| NR2F6 | 0.479 | Down | 6.157E-05 | nucleus |
| TYMP | 1.324 | Up | 5.8001E-05 | nucleus |
| TFRC | 2.177 | Up | 1.9314E-06 | plasma membrane |
